# Prevalence of Risk Factors for the Metabolic Syndrome in the Middle Income Caribbean Nation of St. Lucia

**DOI:** 10.1155/2014/501972

**Published:** 2014-09-22

**Authors:** Colleen O'Brien Cherry, Elizabeth Serieux, Martin Didier, Mary Elizabeth Nuttal, Richard J. Schuster

**Affiliations:** ^1^Center for Global Health, University of Georgia, Athens, GA, USA; ^2^Department of Health Promotion and Behavior, College of Public Health, University of Georgia, Athens, GA, USA; ^3^Department of Internal Medicine, Tapion Hospital, P.O. Box 1780, Castries, Saint Lucia

## Abstract

The objective of this research was to measure the presence of metabolic syndrome risk factors in a sample population in the middle income Caribbean nation of St. Lucia and to identify the demographic and behavioral factors of metabolic syndrome among the study participants. Interviews and anthropometric measures were conducted with 499 St. Lucians of ages 18–99. Descriptive statistics were used for the analysis. Fifty-six percent of females and 18 percent of males had a waist size equal to or above the indicator for the metabolic syndrome. Behavioral risk factors such as sedentary lifestyle, smoking, and alcohol consumption varied by gender. Thirty-six percent of women and 22% of men reported a sedentary lifestyle and 43% of women and 65% of men reported any alcohol consumption. More research should be done to determine the cultural norms and gender differences associated with modifiable risk behaviors in St. Lucia.

## 1. Introduction

There is an alarming increase in the rate of noncommunicable diseases (NCDs) in low and middle income countries where 4 out of 5 deaths occur due to NCDs [1]. The prevalence of metabolic syndrome is increasing globally and particularly in middle income countries. Metabolic syndrome is defined as a concurrence of disturbed glucose and insulin metabolism, abdominal fat, dyslipidemia, and hypertension [2]. Metabolic syndrome increases the risk of diabetes and cardiovascular disease (CVD), both of which are emerging as global epidemics [3]. Rates of obesity (one of the primary drivers of metabolic syndrome), sedentary lifestyle, and urbanization are increasing globally in countries undergoing an epidemiological and nutrition transition and are posited as contributors to the growth in cardiometabolic diseases [4]. Since low and middle income countries are often faced with a double burden of communicable and chronic diseases, these countries will face a tremendous challenge in trying to mitigate the impact of the growing burden of chronic diseases while simultaneously trying to make economic and social progress. Middle income countries in particular are often ignored in deference to the problems faced by high and low income countries. This report addresses the increasing prevalence of metabolic syndrome risk factors in the middle income country of St. Lucia.

### 1.1. Economic and Health Transitions in the Caribbean

Obesity, a risk factor associated with the metabolic syndrome, is on the rise in the Caribbean where many countries are experiencing economic and health transitions [5]. These socioeconomic transitions often include lifestyle changes, such as changes in physical activity and food consumption patterns [6, 7]. St. Lucia's relatively recent transition from an agricultural to a service sector and tourism based economy was prompted by policy changes that disturbed their foothold in banana exports and by the eradication of infectious diseases like schistosomiasis which spurred more foreign visitation to the island and a growing tourism industry [8]. Hruschka et al. [9] have noted that populations are particularly vulnerable to develop obesity during periods of such rapid lifestyle transition [9].

### 1.2. Indicators for Metabolic Syndrome

Physical inactivity (referred to as sedentary lifestyle herein), obesity, smoking, and alcohol intake are thought to be the major modifiable lifestyle and behavioral risks associated with metabolic syndrome [10]. Research has focused on associations between rates of metabolic syndrome and sedentary lifestyle, SES (socioeconomic status) and SEP (socioeconomic position), gender, age, race/ethnicity, and education. One study found that of all of the examined lifestyle factors physical activity was the most strongly associated with a lower risk of having the metabolic syndrome [11]. Since so many of the risk factors for metabolic syndrome are lifestyle related, cultural behaviors must be taken into account in developing medical interventions [12]. However, most studies have been done with white participants and little is understood about whether SEP and metabolic syndrome are associated with particular races or ethnicities. Furthermore, although it has been identified that there are higher associations of metabolic syndrome and SEP factors in women, it is still not known which risk factors of metabolic syndrome are associated most strongly with gender differences. The objectives of this analysis were threefold: (1) to determine the proportion of the study sample that has risk factors for metabolic syndrome, (2) to determine whether risk factors vary by gender, and (3) to identify demographic and behavioral factors associated with indicators for metabolic syndrome in the study population.

## 2. Methods

### 2.1. Research Setting

St. Lucia is an ideal site for looking at prevalence and risk factors of metabolic syndrome due to its status as a middle income country with a characteristic changing economy and increasing urbanization, prompting rapid lifestyle transitions throughout the population. St. Lucia has a majority Afro-Caribbean population of 161,557 [13]. The island is divided into eleven districts, but the most heavily populated area is the capital city of Castries. Castries is located in the north of the island and serves as the business and administrative center as well as being home to 28% of the population [13]. St. Lucians are mostly rural, but the 28% urban population is growing rapidly with the increase in income from tourism and the services industry in the districts of Castries and Gros Islet.

### 2.2. Study Sample

Data collection was carried out during the months of May, June, and July, 2011. We used a convenience sample of St. Lucians who visited one of four of the island's hospitals and clinics within the urban center of Castries. The study sample was 499 St. Lucians of ages 18–99. All males and females above the age of 18 who visited one of the four study sites were included in the study. Outpatients and visitors were approached and interviewed in person at the following four sites: Tapion Hospital (private hospital), Victoria Hospital (public hospital), Rodney Bay Medical Center, and EMCare Medical Center. The interviews were conducted in English and/or* Kweyol*.

### 2.3. Questionnaire

The questionnaire was read to each participant and the interviewer recorded their responses on paper. The questionnaire included self-reported behavioral information on smoking, alcohol consumption, exercise, dietary habits, and demographic information, such as age, gender, address, and ethnicity. Dietary habits were not included in this report since it was decided that the dietary data was out of the scope of this inquiry. The participant's height, weight, and waist circumference were also measured at the time of the interview. Since the project was deemed “quality improvement” (QI), the University of Georgia Office of Sponsored Programs determined that IRB consent was not required. However, for the requirements of QI projects at the Tapion Hospital, there was a consent form which explained the project and was signed by the participants who were recruited at this location. This consent form gave researchers the permission to interview the participants, perform biometric measurements, and access the lipid profiles and fasting glucose levels from current lab reports (if available), in order to conduct the screening for metabolic syndrome indicators at Tapion Hospital. Lipid profiles and fasting glucose were only available for 47 of the total participants and included data from their most recent lab reports at the Tapion Hospital site.

### 2.4. Analysis

Interview data, biometric measurements, lipid profiles, and fasting glucose levels were entered into a spreadsheet and analyzed using descriptive statistics to determine averages and gender differences in the data. We adhered to the National Institutes of Health National Heart Lung, and Blood Institute (NIH/NHLBI) indicators of the metabolic syndrome. The NIH/NHLBI list five conditions as metabolic risk factors: central adiposity, high triglyceride levels, low HDL cholesterol levels, high blood pressure, and high fasting blood sugar [14]. According to NHLBI guidelines [14], metabolic syndrome is diagnosed if three criteria are met. These criteria include a waist circumference greater than 40 (in.) for males or 35 (in.) for females, triglyceride level of 150 mg/dL or higher, HDL less than 40 (mg/dL) for males or less than 50 (mg/dL) for females, a blood pressure of 130/85 mm Hg or higher, and a fasting glucose greater than or equal to 110 (mg/dL) [14]. The data included in this analysis consisted of three out of five of these indicators and the lifestyle associated risk factors of alcohol consumption, exercise, and smoking.

## 3. Results

The data are stratified by gender since the NIH/NHLBI indicators for metabolic syndrome are different for women and men and since one of our objectives was to determine differences in risk factors between women and men. Demographic and behavioral characteristics of the participants are presented in Table 1. Women comprised 349 of the 499 study participants. African ancestry made up over half of the women's and men's sample (see Table 1). The largest percentage of women (47%) and men (43%) live in Castries with Gros Islet making up the second largest district of residence, both of which are growing urban districts. Forty-three percent of women reported drinking any alcohol compared to 65% of men. Thirty-six percent of women and 22% of men reported a sedentary lifestyle. Only 1% of women reported smoking while 13% of men reported smoking.

Table 2 contains the descriptive statistics of the metabolic risk factors. Of note is that only a few cases had all the continuous data present (4 for females and 3 for males). The variable estimated weight was included employing the BMI formula and the BMI and height values. Although BMI is not included as a measure in the NIH/NHLBI indicators of the metabolic syndrome, BMI is often used as a biometric measure since it is the most practical way to define obesity in a clinical setting, so we include it here as a biological indicator of health. Women had a higher mean estimated BMI (28.78) than men (26.52). According to the WHO, a BMI of 25 or above is considered overweight, 25–29.99 is considered “preobese,” and a BMI of 30 or above is considered obese. Women's fasting glucose was 89.47 which was lower than men's fasting glucose of 95.59. However, neither women nor men had a fasting glucose considered as an indicator for metabolic syndrome. Women's HDL was 55.09 which was higher than the men's mean HDL of 46.39. A higher HDL is associated with a decreased risk of CVD. Mean waist circumference for women (36.14 in) was higher than men (35.79 in). Women's average waist circumference is indicative of central adiposity and exceeds the criteria for metabolic syndrome.

Table 3 examines the frequency of NIH/NHLBI defined metabolic indicators stratified by gender to highlight differences between females and males. Due to missing data points, the indicators vary in number of cases included in the frequency. The most prevalent metabolic indicators for women were waist circumference and low HDL (which is an indicator for metabolic syndrome). The most prevalent indicators for men were fasting glucose and low HDL.

## 4. Discussion

This study investigated the presence of metabolic syndrome risk factors and identified gender variation in biological and behavioral risk factors. Women had higher rates of sedentary lifestyle and lower HDL and a higher average waist circumference and BMI (indicative of obesity) than men in the study. Fifty-five percent of the women in our study population have HDL levels within the NIH/NHLBI range of metabolic syndrome indicators and 56% have a waist circumference that falls within this range, placing them at risk for developing metabolic syndrome.

Our findings are in line with previous studies by Loucks et al. [15] and Zeljko et al. [16] who also found that there appear to be gender differences in risk for metabolic syndrome with women at higher risk. As suggested by Loucks et al. [15], this could be associated with gender differences such as parity or the number of live births that women have which may lead to increased central adiposity. Obesity-related effects on social mobility have also been posited with women absorbing more of the psychosocial risks, increasing stigma or increasing depressive symptoms, all of which has been associated with poor metabolic outcomes [17].

Thirty-six percent of women in our sample reported sedentary lifestyle compared with 22% of men. With St. Lucia's recent economic transition, people are now working service industry jobs instead of agricultural jobs, causing them to be more sedentary. The link between exercise and reduced risk of having the metabolic syndrome has been well established in the literature and it is consistent with previous findings by Zhu et al. [11] who found that the risk of having the metabolic syndrome is substantially lower in individuals who are physically active.

The World Health Organization (WHO) reports that 74% of St. Lucia's population is overweight or obese [18]. Of our sample, 31% had BMI of 25 or over indicating overweight or obese. This discrepancy cannot be readily explained as this was a convenience sample and the data collection process used by WHO is not available. St. Lucia's transition to tourism has prompted economic and behavioral changes throughout the population which may help to explain high levels of overweight and obesity in the population. Ongoing ethnographic research with this project team in St. Lucia is underway and preliminary analysis reveals that many St. Lucians feel stressed with the recent urbanization and economic changes in their country. People have moved to urban centers away from their familiar social support system which may cause both social and economic strains [19]. Psychosocial stress is associated with an increased risk for metabolic syndrome [20]. Stress can trigger extra caloric intake in the form of high fat and high carbohydrate foods or alcohol as a means of coping. Similar dietary changes have been reported in the Yucatan Peninsula of Mexico where populations have experienced a nutrition transition toward western diets and reduced physical activity levels, resulting in higher rates of obesity in adults [21].

Obesity is rising worldwide and at an especially fast rate in middle income countries. Its causes are complex and include shifting cultural norms about fat acceptance and body size, increased caloric intake, decreased caloric expenditure, and less nutritional foods [4]. Ongoing ethnographic research in St. Lucia reveals that women tend to accept larger body size than a North American ideal. Preliminary data also reveals that the diet of St. Lucians has changed significantly, with an increase in prepackaged convenience foods and sugar-sweetened drinks and less fresh fruits and vegetables [19]. A recent value added tax of 15% on all products and services has caused further economic strain, and individuals have complained about the high cost of eating healthy and more traditional and healthier foods like fruits, vegetables, and fish [19].

This is the first study of its kind in St. Lucia to estimate the frequency of metabolic syndrome indicators in a sample of the population. This study provides baseline data to develop further hypotheses on metabolic syndrome risk behaviors and clearly demonstrates the need for a more comprehensive analysis of these risk factors in St. Lucia and other middle income countries.

### 4.1. Limitations

The study sample was not a probability sample and therefore may not be representative or generalizable to the population of St. Lucia. The sample was opportunistic since it only included individuals who visited one of the four medical centers and the sample was more heavily populated by women than men. Forty-six percent of the study population lives in Castries, the urban capital which could also have an effect on the results with certain risk behaviors more prevalent in urban areas. Although the four selected medical centers are all located in the north of the island, the sample demographics indicate that the study participants came from all over the island since the centers draw individuals from all over to use the facilities. It might, however, be more fruitful in the future to conduct this same type of survey at different points in various districts around the island to obtain a more representative population sample. There is no single universally applicable standard measure that exists for metabolic syndrome or for obesity. Three of the most commonly used measures for obesity are BMI and waist circumference and waist to hip ratio. The usefulness of each measure varies and is highly contested, in particular with non-Caucasian populations, due to differences in body composition and ectopic fat deposition [22]. The behavioral data on modifiable lifestyle risks of smoking, exercise, and diet relied on self-reports which may lead to inaccurate estimation. We also had missing data points for HDL and RBS, so we were not able to test prevalence of metabolic syndrome indicators in the full sample.

## 5. Conclusions

Among study participants, women had a higher prevalence of metabolic syndrome risk factors including waist circumference, low HDL, BMI, and sedentary lifestyle. Explanations for why there are gender differences in the prevalence of metabolic syndrome risk factors are beyond the scope of this study. However, other international studies on metabolic syndrome have suggested that socioeconomic factors play a major role in increasing risks and that cultural beliefs and norms are immensely important in middle income countries like St. Lucia [23]. More research should be done to determine the cultural norms that may drive an increase in modifiable risk behaviors in St. Lucia. Further work should also focus on how gender norms and cultural and economic changes relate to behavioral risk factors for the metabolic syndrome. In order to mitigate the prevalence of metabolic syndrome and CVD and other associated noncommunicable diseases, the biggest lifestyle and behavioral health threats need to be identified and addressed through clinical interventions, population wide prevention programs, and policy interventions.

## Figures and Tables

**Table 1:** Frequency of demographic and behavioral variables. (*N* = 499).

Female			Male		
Variables	Freq.	%	Variables	Freq.	%
Ancestry			Ancestry		
African	194	55.6	African	81	54.0
Mixed^1^	135	38.7	Mixed ^1^	55	36.7
Other^2^	15	4.3	Other^2^	12	8.0
Alcohol			Alcohol		
Yes	150	43.0	Yes	98	65.3
No	193	55.3	No	52	34.7
District			District		
A La Raye	32	9.2	A La Raye	13	8.7
Castries^3^	164	47.0	Castries	64	42.7
Choiseul	5	1.4	Choiseul	1	0.7
Dennery	20	5.7	Dennery	5	3.3
Gros Islet^3^	101	28.9	Gros Islet	51	34.0
Laborie	2	0.6	Laborie	0	0.0
Micoud	6	1.7	Micoud	2	1.3
Soufriere	9	2.6	Soufriere	3	2.0
Vieux Fort	10	2.9	Vieux Fort	11	7.3
Exercise			Exercise		
Sedentary	127	36.4	Sedentary	33	22.0
Mild	90	25.8	Mild	35	23.3
Moderate	59	16.9	Moderate	36	24.0
Intense	66	18.9	Intense	45	30.0
Smoking			Smoking		
Yes	4	1.1	Yes	19	12.7
No	337	96.6	No	129	86.0

Female *n* = 349. Male *n* = 150. Differences between categories total count and the total gender sample size indicative of missing data.

^1^Mixed indicates African and Indian descent.

^2^Other indicates Indo-Caribbean, Asian-Indian, or various other descents.

^3^Urban district.

**Table 2:** Descriptive statistics for metabolic syndrome indicators.

Female				Male			
Variables	Mean	St. dev.	*n*	Variables	Mean	St. dev.	*n*
BMI	28.8	6.27	342	BMI	26.5	5.03	149
Fasting glucose	89.5	76.97	43	Fasting glucose	95.6	65.52	25
HDL	55.1	19.53	33	HDL	46.4	9.17	23
Waist	36.1	5.69	345	Waist	35.8	5.36	149

Female *n* = 349; valid *n* = 4. Male *n* = 150; valid *n* = 3. HDL: high-density lipoprotein.

**Table 3:** Frequency of metabolic syndrome indicators.

Female				Male			
Variables	Freq.	%	Valid cases	Variables	Freq.	%	Valid cases
HDL	18	54.5	33	HDL	5	21.7	23
RBS	11	25.6	43	RBS	9	36.0	25
W	192	55.7	345	W	27	18.1	149

Female *n *= 349. Male *n* = 150. HDL: high-density lipoprotein. RBS: fasting glucose greater than or equal to 110 (mg/dL). W: waist circumference greater than 40 (in.) for males or 35 (in.) for females. Low HDL is less than 50 (mg/dL) for females and less than 40 (mg/dL) for males.
